# Identification of miRNAs in a Liver of a Human Fetus by a Modified Method

**DOI:** 10.1371/journal.pone.0007594

**Published:** 2009-10-26

**Authors:** Dong Liu, Jing Fan, Manxue Mei, Sigurdur Ingvarsson, Huiping Chen

**Affiliations:** 1 Department of Medical Genetics, Tongji Medical College, Huazhong University of Science and Technology, Wuhan, Hubei, China; 2 Department of Feed Science, Hubei Key Laboratory of Animal Nutrition and Feed Science, Wuhan Polytechnical University, Wuhan, Hubei, China; 3 Institute for Experimental Pathology and Faculty of Medicine, University of Iceland, Keldur, Reykjavik, Iceland; Texas A&M University, United States of America

## Abstract

**Background:**

miRNAs are 17–25 nucleotides long RNA molecules that have been found to regulate gene expression in human cells. There are studies showing that different groups of miRNAs are involved in development of different tissues. In hepatocytes there are reported particular types of miRNAs that regulate gene expression.

**Methods:**

We established a human fetal liver cDNA library by a modified cloning protocol. Then plasmid isolation from the colonies was performed. After sequencing and database searching, the miRNAs were recognized. RT-PCR and sequencing were carried out to validate the miRNAs detected. Real-time PCR was used to analyze the expression of each miRNA.

**Results:**

One novel miRNA was discovered, together with another 35 previously-known miRNAs in the fetal liver. Some of them existed in variants. The miRNAs identified were validated by RT-PCR and sequencing. Quantitative analysis showed that they have variable expression.

**Conclusion:**

Our results indicate that a special group of miRNAs may play an important role in fetal liver development in a synergistic manner.

## Introduction

The epigenetic silencing of genes after transcription can be caused by either exogenous small interfering RNAs (siRNAs) or endogenous microRNAs (miRNAs). The miRNAs are non-coding RNAs which repress translations of target mRNAs. The miRNA genes encode for primary miRNAs (pri-miRNAs). These pri-miRNAs are trimmed into approximate 70 nucleotides of hairpin structures, called precursor miRNAs (pre-miRNAs), by the RNase III type protein, Drosha, in the nucleus. The pre-miRNAs are then transported to the cytoplasm by Exportin-5 and are cleaved to 22 nucleotides of mature miRNAs by Dicer enzymes, another RNase type III [1].

Studies show that miRNAs play a bigger role in the important biological processes. Some miRNAs, e.g., let-7 and lin-4, can regulate the timing of early and late larval developmental transition in *Caenorhabditis elegans* [2], [3]. In plants, some miRNAs regulate flowering, leaf development and embryonic patterning [4]–[6]. Furthermore, miR-14 and bantam are found to regulate apoptosis, growth and fat metabolism in *Drosophila* [7], [8].

The miRNAs play important roles in development and differentiation of human organs [9]–[12]. Some of them exhibit tissue-specific expression [13]. To date more than 850 human miRNAs have been identified (miRBase database) (http://microrna.sanger.ac.uk/index.shtml). Detection of additional miRNAs can be expected in the future. The usual way to find novel miRNAs is to use a miRNA cloning method [14], which can also identify variants for some miRNAs [15]. The miRNA variants may have different target genes, compared to wildtype miRNAs.

The liver is a crucial human organ, in which miRNAs should be involved in regulation of hepatocyte growth and development. The involvement of miRNAs in fetal liver and adult liver may be different, as miRNA expression also shows developmental stage specificity [16]. Hitherto, only one article has reported miRNA identification in human fetal liver: the authors did not specify the age of the fetus [15].

In this study we identified 36 miRNAs from the liver of a human fetus of 27 weeks by a modified cloning method. Of these miRNAs, 35 were identical or variant to those described previously, and one was novel.

## Results and Discussion

### Detection of 36 miRNAs from the cDNA library and confirmation of miRNA expression by RT-PCR and sequencing

A cDNA library for small RNAs (≤200 nt) was constructed by a modified cloning method. Based on a previous study [15], we modified the 5′ linker and RT primer. The 5′ linker and RT primer we used are shorter than those reported before. They worked well and cost less. This method has advantages that the microarray method lacks, for example in the discoveries of novel and variant miRNAs.

**Figure 1:**
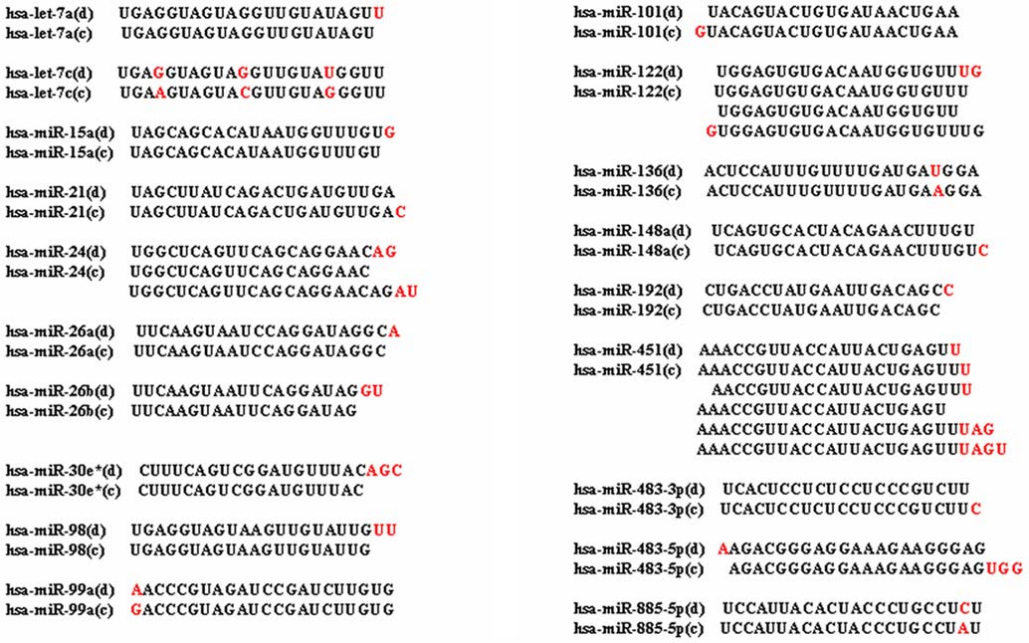
Variants detected for some miRNAs. The difference between miRNAs from the database (d) and the cloned (c) is highlighted in red.

**Figure 2:**
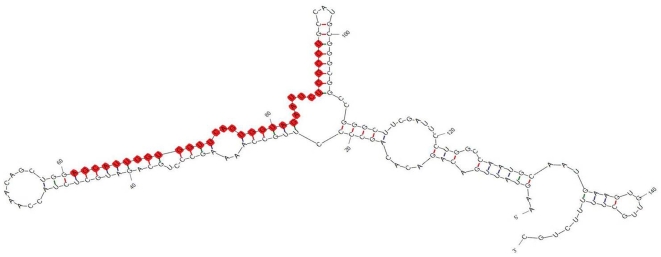
Predicted secondary structure of primary miRNA for the novel miRNA identified. The bases highlighted in red represent mature miRNA.

The clones were picked up from the LB plates for analysis. A total of 143 clones were subsequently examined by DNA sequencing and database searching. Several kinds of cellular RNA fragments were detected (Table 1). More than 20% of the cloned RNAs were degraded products of abundant RNAs such as rRNA and mRNA, and 111 clones were identified as miRNAs. As shown in Table 2, the majority are known miRNAs, and only one is novel miRNA, which was identified by using the flanking sequences to predict secondary structure through Mfold. As regards the previously-known miRNAs, some were detected as variants of miRNAs in the miRNA database (Fig. 1 and Table 2). The novel miRNA is 33 bp, larger than the common ones. It did not show homology with any known miRNA or other sources of RNAs. However, the 33 bp of miRNA has an identical sequence in the human genome and has a typical hairpin which was predicted using Mfold (Fig. 2).

**Table 1:** Composition of small RNA populations cloned from fetal liver.

RNA class	Number
**miRNA**	**111**
**mRNA**	**12**
**rRNA**	**8**
**Match with genome** a	**10**
**Unknown** b	**2**
**Total**	**143**

a Sequences that do not form miRNA-specific hairpin precursors but only match with genomic sequences.

b Sequences that do not match with any genomic sequences.

**Table 2:** miRNAs identified in fetal liver.

miRNA	Sequence (5′–3′)	No. clones	Size(nt)
**hsa-let-7a**	**UGAGGUAGUAGGUUGUAUAGU**	**1**	**21**
**hsa-let-7b**	**UGAGGUAGUAGGUUGUGUGGUU**	**1**	**22**
**hsa-let-7c**	**UGAAGUAGUACGUUGUAGGGUU**	**1**	**22**
**hsa-let-7d**	**AGAGGUAGUAGGUUGCAUAGUU**	**1**	**22**
**hsa-let-7f**	**UGAGGUAGUAGAUUGUAUAGUU**	**2**	**22**
**hsa-let-7g**	**UGAGGUAGUAGUUUGUACAGUU**	**1**	**22**
**hsa-miR-15a**	**UAGCAGCACAUCAUGGUUUGU**	**2**	**21**
**hsa-miR-19a**	**UGUGCAAAUCUAUGCAAAACUGA**	**1**	**23**
**hsa-miR-19b**	**UGUGCAAAUCCAUGCAAAACUGA**	**2**	**23**
**hsa-miR-21**	**UAGCUUAUCAGACUGAUGUUGA**	**1**	**22**
	**UAGCUUAUCAGACUGAUGUUGAC**	**1**	**23**
**hsa-miR-23a**	**AUCACAUUGCCAGGGAUUCCC**	**1**	**21**
**hsa-miR-24**	**UGGCUCAGUUCAGCAGGAACAG**	**2**	**22**
	**UGGCUCAGUUCAGCAGGAAC**	**1**	**20**
	**UGGCUCAGUUCAGCAGGAACAGAU**	**1**	**24**
**hsa-miR-26a**	**UUCAAGUAAUCCAGGAUAGGC**	**1**	**21**
**hsa-miR-26b**	**UUCAAGUAAUUCAGGAUAG**	**1**	**19**
	**UUCAAGUAAUUCAGGAUAGGU**	**1**	**21**
**hsa-miR-27a**	**UUCACAGUGGCUAAGUUCCGC**	**1**	**21**
**hsa-miR-27b**	**UUCACAGUGGCUAAGUUCUGC**	**1**	**21**
**hsa-miR-30d**	**UGUAAACAUCCCCGACUGGAAG**	**1**	**22**
hsa-miR-30e^*^	**CUUUCAGUCGGAUGUUUAC**	**1**	**19**
**hsa-miR-98**	**UGAGGUAGUAAGUUGUAUUG**	**1**	**20**
**hsa-miR-99a**	**GACCCGUAGAUCCGAUCUUGUG**	**1**	**22**
**hsa-miR-101**	**GUACAGUACUGUGAUAACUGAA**	**1**	**22**
**hsa-miR-103**	**AGCAGCAUUGUACAGGGCUAUGA**	**1**	**23**
**hsa-miR-122**	**UGGAGUGUGACAAUGGUGUU**	**4**	**20**
	**UGGAGUGUGACAAUGGUGUUU**	**7**	**21**
	**UGGAGUGUGACAAUGGUGUUUG**	**39**	**22**
	**GUGGAGUGUGACAAUGGUGUUUG**	**1**	**23**
**hsa-miR-125a-5p**	**UCCCUGAGACCCUUUAACCUGU**	**1**	**22**
**hsa-miR-126**	**UCGUACCGUGAGUAAUAAUGCG**	**2**	**22**
**hsa-miR-136**	**ACUCCAUUUGUUUUGAUGAAGGA**	**1**	**23**
**hsa-miR-144**	**UACAGUAUAGAUGAUGUACU**	**1**	**20**
**hsa-miR-148a**	**UCAGUGCACUACAGAACUUUGU**	**2**	**22**
	**UCAGUGCACUACAGAACUUUGUC**	**1**	**23**
**hsa-miR-192**	**CUGACCUAUGAAUUGACAGCC**	**1**	**21**
	**CUGACCUAUGAAUUGACAGC**	**2**	**20**
**hsa-miR-194**	**UGUAACAGCAACUCCAUGUGGA**	**3**	**22**
**hsa-miR-410**	**AAUAUAACACAGAUGGCCUGU**	**1**	**21**
**hsa-miR-451**	**AAACCGUUACCAUUACUGAGU**	**1**	**21**
	**AAACCGUUACCAUUACUGAGUU**	**1**	**22**
	**AACCGUUACCAUUACUGAGUUU**	**1**	**22**
	**AAACCGUUACCAUUACUGAGUUU**	**4**	**23**
	**AAACCGUUACCAUUACUGAGUUUAG**	**1**	**25**
	**AAACCGUUACCAUUACUGAGUUUAGU**	**2**	**26**
**hsa-miR-483-3p**	**UCACUCCUCUCCUCCCGUCUUC**	**1**	**22**
**hsa-miR-483-5p**	**AGACGGGAGGAAAGAAGGGAGUGG**	**1**	**24**
**hsa-miR-885-5p**	**UCCAUUACACUACCCUGCCUAU**	**1**	**22**
**hsa-miR-novel**	**AGCAUUGGUGGUUCAGUGGUAGAAUUCUCGCCU**	**2**	**33**
**total**	**/**	**111**	**/**

A total of 36 miRNAs were detected in this fetal liver, especially the liver-specific miRNAs, including miR-122, miR-148, miR-192 and miR-194 [17], were all identified (Table 2). Compared to the report by Fu et al. [15], different types of miRNAs were detected in our study. The first reason for this difference could be that the livers are at different stages of development, and different miRNAs may play roles in specific periods. The second reason could be that the number of clones examined in our study is larger than in that study. The third explanation could be that a bias for clonal selection emerged when we picked up the colonies on the LB plates.

In our study, most of members of the let-7 family were identified (Table 2); these were however, not detected at all in the study of Fu et al [15]. The let-7 family acts as a tumour suppressor and plays a critical role in cell-cycle control with respect to differentiation and tumourigenesis [18]. The expression of the let-7 family detected in the middle of the pregnancy suggests a certain level of negative regulation of cell growth in the 27-week fetal liver.

**Figure 3:**
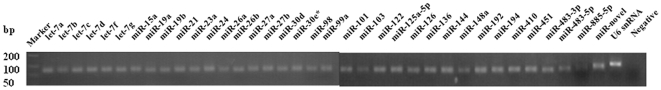
RT-PCR for miRNAs. Positive control is U6 small nuclear RNA. H2O serves as negative control.

**Figure 4:**
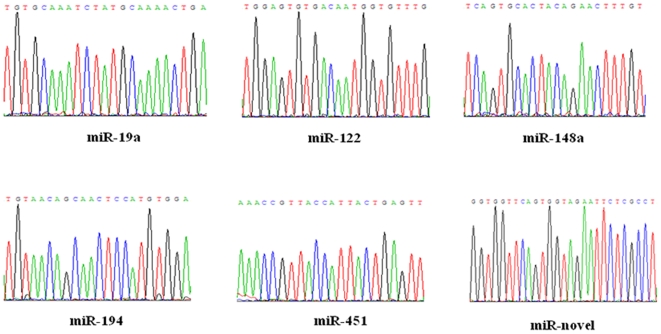
Sequencing of the RT-PCR products for 6 miRNAs.

The miRNAs identified above were confirmed by using RT-PCR and sequencing. Figure 3 shows each miRNA was amplified, suggesting that they were stably expressed in the fetal liver. The RT-PCR products were then sequenced and all miRNA sequences were found correct (Fig. 4).

### Comparison of miRNA expression using real-time PCR

**Figure 5:**
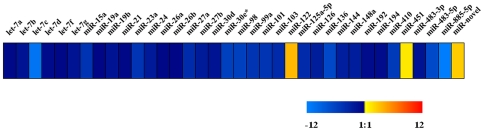
Expression profiles of 36 miRNAs by real-time PCR. The relative expression values ranged from −12 log_2_ to +12 log_2_.

In order to ascertain their relative expression level, a real-time PCR was carried out, and we found that miR-122 (p<0.0001), miR-451 (p = 0.0057) and miR-novel (p = 0.0006) have a higher level compared to others (Fig. 5), suggesting that these miRNAs play an important role in development and differentiation of the liver at 27 weeks. However, the other three liver-specific miRNAs (miR-148, miR192 and miR-194) exhibited moderate expression levels (Fig. 5). A previous study of an adult liver reported high expression of miR-122 and miR-194, and moderate expression of miR-148 and miR-192 [17], which is similar to our study (Fig. 5), indicating that these liver-specific miRNAs are important for the two developmental stages of the liver. In that study, let-7a, let-7b, miR-21 and miR-27b also showed a moderate expression level [17], which is in line with this study (Fig. 5).

### Analysis of potential targets for the miRNAs

A prediction of target genes for the known miRNAs was undertaken using software for miRNA targets version 5 and TargetScan 5.1, and their target genes in the liver were predicted (Table 3). Interestingly, some miRNAs act on the same target genes (Table 3), suggesting that there is synergy between these miRNAs during hepatic development. The highest expression level was detected for miR-122, whose target genes include PINX1_HUMAN (Table 3). This gene may inhibit cell proliferation and act as tumour suppressor [19]. It has been suggested that inhibition of this gene may promote hepatic growth.

**Table 3:** Prediction of target genes of miRNAs identified.

miRNAs	Target genes in human liver
**hsa-let-7a**	**PRDX5**
**hsa-let-7b**	**PRDX5, NP_060880.3, CPT1A**
**hsa-let-7c**	**NP_060880.3, CPT1A**
**hsa-let-7d**	**GJB1**
**hsa-let-7f**	**CPT1A, TMEM59**
**hsa-let-7g**	**PFKFB1, PRDX5**
**hsa-miR-15a**	**GLS2, PFKFB1**
**hsa-miR-19a**	**PSG1, DLC1**
**hsa-miR-19b**	**PSG1**
**hsa-miR-21**	**PHKA2, CCL20, GJB1, PYGL**
**hsa-miR-23a**	**PTP4A2, ARG1**
**hsa-miR-24**	**PHLDA2, DNASE1L3, PSG5, PINX1_HUMAN**
**hsa-miR-26a**	**TMEM59**
**hsa-miR-26b**	**TMEM59**
**hsa-miR-27a**	**PFKL, PFKFB1, COX8A, NP_060880.3**
**hsa-miR-27b**	**PFKL, PFKFB1, COX8A, NP_060880.3**
**hsa-miR-30d**	**N.D.**
hsa-miR-30e^*^	**PRDX5**
**hsa-miR-98**	**PRDX5, Q6SA06_HUMAN**
**hsa-miR-99a**	**STARD13, COX6A1**
**hsa-miR-101**	**PFKFB1**
**hsa-miR-103**	**PHKG2, Q86U10_HUMAN, GLS2**
**hsa-miR-122**	**PKLR, ALPL, PINX1_HUMAN, PSG5**
**hsa-miR-125a-5p**	**NR5A2, STARD13**
**hsa-miR-126**	**CSTB**
**hsa-miR-136**	**PBLD, PYGL, STARD13, TMEM59**
**hsa-miR-144**	**ALDOB, CCL20**
**hsa-miR-148a**	**PKLR, PHKG2**
**hsa-miR-192**	**PTP4A3, TM4SF4, HAMP, COX7A2**
**hsa-miR-194**	**N.D.**
**hsa-miR-410**	**PSG1, TMEM59, CLEC4M**
**hsa-miR-451**	**TMEM59**
**hsa-miR-483-3p**	**OSGIN1, GJB1, DLC1**
**hsa-miR-483-5p**	**PTP4A3, HAMP, GFER**
**hsa-miR-885-5p**	**Q86U10_HUMAN**
**hsa-miR-novel**	**LEAP2, DLC1**

N.D., no data.

A novel miRNA was also detected in this liver organ, which is larger than the previously described miRNAs. Its predicted target genes in the liver organ are LEAP2 (liver expressed antimicrobial peptide 2) and DLC1 (deleted in liver cancer 1) (Table 3). It is suggested that DLC1 is a candidate tumour suppressor gene for human liver cancer, as well as for prostate, lung, colorectal and breast cancers [20]. Larger miRNAs (dan-mir-289: 35 bp) have been identified in *Drosophila* (miRBase database). These have not been detected in human before.

Interestingly, the variants for some miRNAs were found, including single-nucleotide substitution and length difference (Fig. 1 and Table 2). Some of them were also analysed by RT-PCR and real-time PCR (Fig. 3 and Fig. 5). Specially, the variants with 3 and 4 bases longer for miR-451 were also analysed using RT-PCR and real-time PCR, and found to have stable expression (data not shown). We speculate that the single-nucleotide substitution is due to the post-transcriptional modifications, e.g. A-to-I editing (identified as A-to-G changes) [21] or the single-nucleotide polymorphism (SNP) in the miRNA genes [22], and length difference to alternative cleavage of the hairpins by the Dicer enzymes, because in the majority of miRNAs the bases added were found to be next to the mature sequences in the hairpin secondary structures. We can exclude that the variants are generated by PCR and/or sequencing errors, because our RT-PCR amplified all of the variants. The length difference has also been identified in the cDNA library for silkworm miRNAs [23]. It is conceivable that these variants have different target genes; thus certain types of miRNAs could inhibit multiple target genes. For example, five variants have been identified for miR-451 (Fig. 1). It can be speculated that these variants act on different target genes compared to the wildtype of miR-451.

### Conclusions

Our results showed that a special group of miRNAs have been expressed in the fetal liver of 27 weeks. These discoveries help shed light on the fine-tuning mechanism of miRNAs in hepatocyte development and differentiation. Further studies are needed in order to identify the exact target genes of these miRNAs.

## Materials and Methods

### Ethics Statement

This research has been approved by the review board of Huazhong University of Science and Technology. We obtained tissue samples with written informed consent from the participant involved in the study. The ethics committee specifically approved that procedure.

### Isolation of small RNA

The liver tissue was obtained from a female fetus of 27 weeks delivered due to severe syndrome of high blood pressure of the mother in Tongji hospital, Wuhan, Hubei, China. The fetus died shortly after delivery. The mother of the fetus had no other diseases than high blood pressure. Small RNA (≤200 nt) was isolated from the liver tissue using a mirVana™ miRNA isolation kit (Ambion, Austin, TX) following the manufacturer's instructions. About fifty milligrams of tissue were used and the small RNA was eluted in 100 µl RNase-free water. The RNA concentration was tested by UV absorbance at 260 nm.

### Establishment and screen of cDNA library

The RNAs above were polyadenylated at 37°C for 30 min in 50 µl reaction volume using ∼1 µg RNA and 5 U poly(A) polymerase (New England Biolabs). Then the Poly(A)-tailed small RNA was purified through phenol/chloroform extraction and ethanol precipitation. A 5′ linker (5′-GGA CAC UGA CAU GGA CUG AAG GAG UAG AAA-3′) was ligated to poly(A)-tailed RNA using T4 RNA ligase (New England Biolabs) and the ligation products were recovered by phenol/chloroform extraction followed by ethanol precipitation. Reverse transcription was conducted using the entire poly(A)-tailed RNA and 1 µg RT primer (5′-CGC TAC GTA ACG GCA TGA CAG TG (T)24-3′) with 200 U of SuperScript III reverse transcriptase (Invitrogen) according to the manufacturer's instructions. The cDNA was amplified for 35 cycles at an annealing temperature of 55°C using primers 5′-GGA CAC TGA CAT GGA CTG AAG GAG TA-3′ and 5′-CGC TAC GTA ACG GCA TGA CAG TG-3′. The PCR products were run on 3% agarose gel with GoldView staining. Then the gel slices with DNA of a size about 110 bp were excised and purified using EasyPure quick gel extraction kit (TransGen Biotech). The DNA fragments were directly cloned into pEASY-T1 vector (TransGen Biotech). Colony PCR was performed using 5′ and 3′ primers, and the clones with PCR products about 110 bp in length were sequenced.

### Validation of miRNA expression by RT-PCR and sequencing

Reverse transcription was carried out using ∼1 µg poly(A)-tailed small RNA and 1 µg of the same RT primer as above with 200 U of SuperScript III (Invitrogen) according to the method described above. Amplification of miRNA was conducted for 35 cycles at an annealing temperature of 55°C using miRNA-specific forward primers (Table 4) and reverse primer (5′-CGC TAC GTA ACG GCA TGA CAG TG-3′). The internal control was U6 small nuclear RNA, because U6 is stably expressed in human tissues. The PCR products were separated on 3% agarose gel with GoldView staining. Then the PCR bands were excised and purified. The purified PCR products were cloned into pEASY-T1 vector (TransGen Biotech), and clones with PCR products were sequenced.

**Table 4:** miRNA specific primers for RT-PCR and real-time PCR.

miRNA	primer
**hsa-let-7a**	TGAGGTAGTAGGTTGTATAGT
**hsa-let-7b**	TGAGGTAGTAGGTTGTGTGGTT
**hsa-let-7c**	TGAAGTAGTACGTTGTAGGGTT
**hsa-let-7d**	AGAGGTAGTAGGTTGCATAGTT
**hsa-let-7f**	TGAGGTAGTAGATTGTATAGTT
**hsa-let-7g**	TGAGGTAGTAGTTTGTACAGTT
**hsa-miR-15a**	TAGCAGCACATAATGGTTTGT
**hsa-miR-19a**	TGTGCAAATCTATGCAAAACTG
**hsa-miR-19b**	TGTGCAAATCCATGCAAAACTG
**hsa-miR-21**	TAGCTTATCAGACTGATGTTG
**hsa-miR-23a**	ATCACATTGCCAGGGATTCC
**hsa-miR-24**	TGGCTCAGTTCAGCAGGAACAG
**hsa-miR-26a**	TTCAAGTAATCCAGGATAGG
**hsa-miR-26b**	TTCAAGTAATTCAGGATAG
**hsa-miR-27a**	TTCACAGTGGCTAAG TTCC
**hsa-miR-27b**	TTCACAGTGGCTAAGTTCTGC
**hsa-miR-30d**	TGTAAACATCCCCGACTGGAAG
hsa-miR-30e^*^	CTTTCAGTCGGATGTTTAC
**hsa-miR-98**	TGAGGTAGTAAGTTGTATTG
**hsa-miR-99a**	GACCCGTAGATCCGATCTTGTG
**hsa-miR-101**	TACAGTACTGTGATAACTGAA
**hsa-miR-103**	AGCAGCATTGTACAGGGCTATG
**hsa-miR-122**	TGGAGTGTGACAATGGTGTTTG
**hsa-miR-125a-5p**	TCCCTGAGACCCTTTAACCTGT
**hsa-miR-126**	TCGTACCGTGAGTAATAATG
**hsa-miR-136**	ACTCCATTTGTTTTGATGAAGG
**hsa-miR-144**	TACAGTATAGATGATGTACT
**hsa-miR-148a**	TCAGTGCACTACAGAACTTTGT
**hsa-miR-192**	CTGACCTATGAATTGACAGC
**hsa-miR-194**	TGTAACAGCAACTCCATGTGG
**hsa-miR-410**	AATATAACACAGATGGCCTGT
**hsa-miR-451**	AAACCGTTACCATTACTGAGTT
**hsa-miR-483-3p**	TCACTCCTCTCCTCCCGTCTT
**hsa-miR-483-5p**	AGACGGGAGGAAAGAAGGGAGTGG
**hsa-miR-885-5p**	TCCATTACACTACCCTGCCTAT
**hsa-miR-novel**	GGTGGTTCAGTGGTAGAATTCTC

### miRNA quantitative analysis using real-time PCR

Real-time PCR was carried out in a Stratagene MX-4000 system with the same primers as detection of miRNA expression by RT-PCR and TransStart SYBR green qPCR supermix (TransGen, Biotech) following the manufacturer's instructions. The 20 µl reactions including 0.5 µl of RT products, 1x TransStart SYBR green qPCR supermix and 0.5 µM forward and reverse primers were incubated at 95°C for 5 min, followed by 40 cycles of 95°C for 10 sec, 55°C for 15 sec and 72°C for 20 sec. The amplification curves (Fig. S1) were examined and no differences of PCR efficiencies between the reactions were seen. Melting curves for each PCR were carefully monitored to avoid non-specific amplification. The U6 small nuclear RNA was used as internal control. Each miRNA was analysed in duplicate. Relative level (RL) of each miRNA expression was calculated with 2^−ΔCt^ method, and the data were presented as log_2_ of RL of target miRNAs. Results were visualized with GENESIS (Alexander Sturn, Institute for Genomics and Bioinformatics, Graz University of Technology).

### Biological software analysis

The small RNA sequences were analysed using BLAST analysis against the human genome and miRNA databases. The secondary structures of RNA precursors were built by using longer genomic sequences of cloned RNAs and the Mfold program (http://mfold.bioinfo.rpi.edu/cgi-bin/rna-form1.cgi).

### Statistical analysis of miRNA expression

A two-tailed *t*-test was used to assess the expression level difference between miRNA and U6.

### Target gene prediction of each miRNA

Target genes for each miRNA were predicted using miRBase Targets Version 5 program (http://microrna.sanger.ac.uk/targets/v5/) and TargetScan 5.1 program (http://www.targetscan.org/).

## Supporting Information

Figure S1 The amplification plots for real-time PCR.
(0.11 MB PPT)
