# Supplementary figures and images for: Identification of miRNAs in a Liver of a Human Fetus by a Modified Method
Source: PLoS One. 2009 Oct 26;4(10):e7594. doi: 10.1371/journal.pone.0007594 (PMC2762743; doi:10.1371/journal.pone.0007594)

## Slide 1
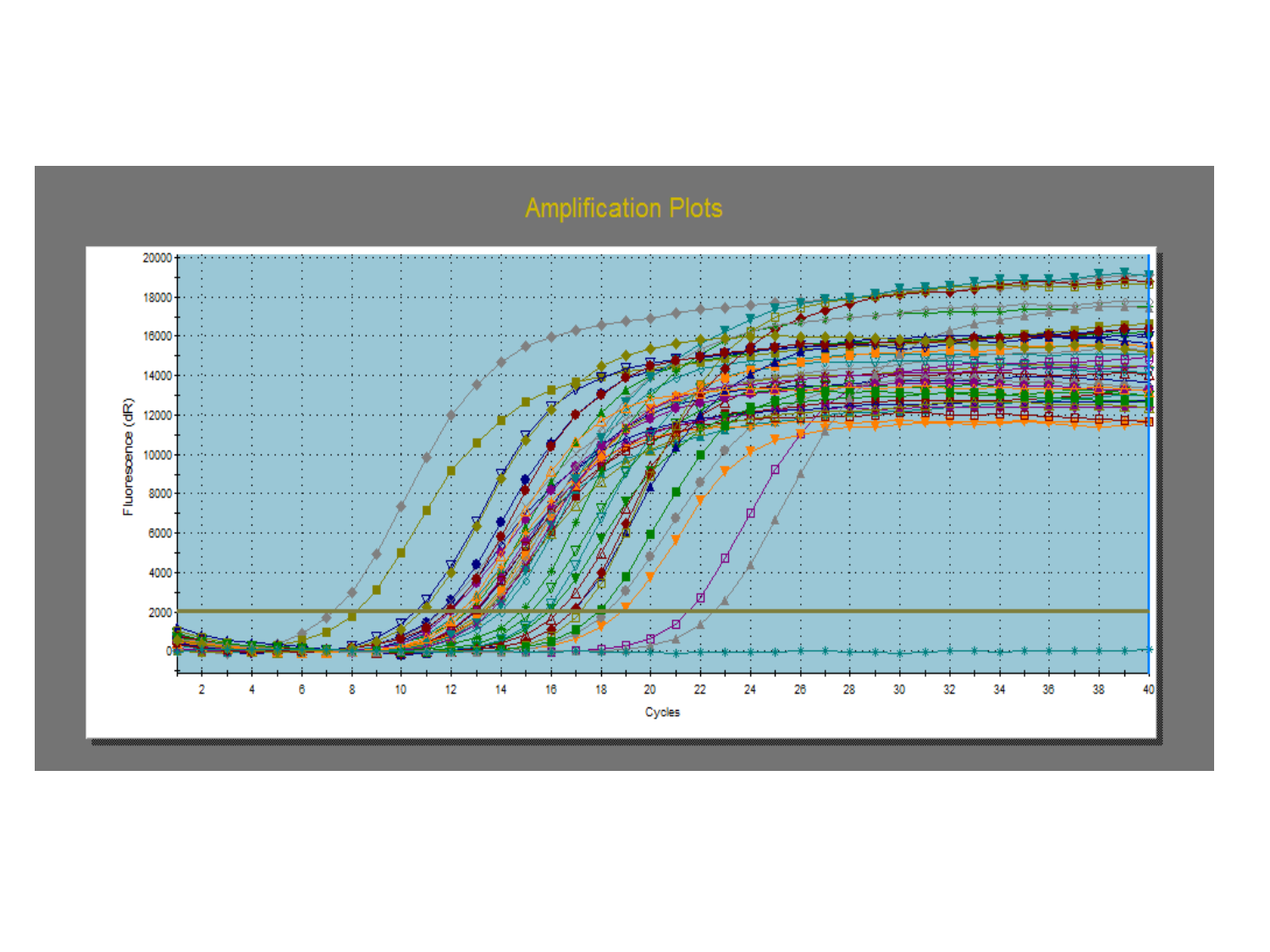

Supplement: Figure S1 — The amplification plots for real-time PCR. (0.11 MB PPT) [file pone.0007594.s001.ppt]
